# Recepteur d'origine nantais contributes to the development of endometriosis via promoting epithelial‐mesenchymal transition of a endometrial epithelial cells

**DOI:** 10.1111/jcmm.16261

**Published:** 2021-01-06

**Authors:** Qin Yu, Jianzhang Wang, Tiantian Li, Xinyue Guo, Shaojie Ding, Xuan Che, Libo Zhu, Yangying Peng, Xinxin Xu, Gen Zou, Xinmei Zhang

**Affiliations:** ^1^ Women's Hospital School of Medicine Zhejiang University Hangzhou China; ^2^ Jiaxing University Affiliated Women and Children Hospital Jiaxing China

**Keywords:** endometriosis, epithelial‐mesenchymal transition, invasion, migration, recepteur d'origine nantais

## Abstract

Endometriosis is a benign, chronic inflammatory disease that commonly occurs in reproductive‐aged women. Epithelial**‐**mesenchymal transition (EMT) of endometrial epithelial cells plays an important role in the development of endometriosis. Recepteur d'origine nantais (RON), a receptor tyrosine kinase, has been reported to promote EMT and progression in tumours. However, whether and how RON mediates the EMT and endometriosis development is not known. Here, we found that RON activation could improve the migratory and invasive capabilities, change cellular morphologies, and decrease expression of E‐cadherin and increase expression of N‐cadherin in endometrial epithelial cells. Inhibition or knockdown of RON expression suppressed the migration and invasion of endometrial epithelial cells. Our studies also indicated that RON played its part in endometrial epithelial cells through protein kinase B (Akt) and mitogen‐activated protein kinase (MAPK) pathways. Treatment with a RON inhibitor could decrease the number of ectopic lesions in a mouse model of endometriosis and mediate expression of EMT markers in endometriotic lesions. These data suggest that RON contributed to endometriosis development by promoting EMT of endometrial epithelial cells. Therefore, RON may be a new therapeutic target for endometriosis.

## 1 INTRODUCTION

Endometriosis is characterized by functional endometrial glands and stroma located outside of the uterine cavity. It is a benign, oestrogen‐dependent and chronic inflammatory gynaecological benign disease that results in dysmenorrhoea, infertility and pelvic pain.^1^, ^2^, ^3^ The incidence of endometriosis is increasing, but its aetiology and pathogenesis are still unclear up to date. Stem cells, epithelial metaplasia, lymphatic metastasis and retrograde menstruation (RM) have been used to explain the cause of endometriosis, but the RM theory is the most popular.^4^, ^5^ Migration and invasion of endometrial cells underpin the RM theory, and these two processes have been demonstrated to be implicated in epithelial**‐**mesenchymal transition (EMT).^6^, ^7^, ^8^, ^9^

During EMT, cells lose their epithelial features and acquire the characteristics of mesenchymal cells.^10^, ^11^ When EMT occurs, the expression of mesenchymal markers (eg N‐cadherin) is increased. However, the expression of epithelial molecular markers (eg E‐cadherin) is decreased.^12^, ^13^ In addition, the expressions of cell polarity (such as Par3) and tight junction (such as Occludin) proteins are decreased, which destroys the epithelial cell polarity and tight junctions between cells.^14^, ^15^, ^16^ As a result, the abilities of migration, invasion and anti‐apoptosis are increased.^10^, ^11^, ^17^ Recently, several scholars have shown that EMT can make the endometrial cells from RM obtain the ability of migration, invasion and anti‐apoptosis. These features are beneficial for endometrial implantation in the abdominal cavity, suggesting an important role of EMT in endometriosis development.^18^, ^19^ Oestrogen and hypoxia are currently considered to be two inducers of EMT for endometrial cells originating from RM, but how EMT is triggered in endometriosis is still unclear.^20^, ^21^

The receptor tyrosine kinase, recepteur d'origine nantais (RON, also known as macrophage‐stimulating protein receptor, MST1R), belongs to the MET family of receptors^22^ and its ligand is macrophage‐stimulating protein (MSP).^23^, ^24^ RON is expressed mainly in various types of human epithelial tissues including endometrial epithelial cells. Overexpression of RON has been proved to be associated with the progression, metastasis, survival and prognosis of various types of cancers including cancer of the pancreas, colon, lung, breast and ovary.^25^, ^26^, ^27^, ^28^ In women with endometriosis, increased expression of RON has also been found in endometriotic lesions, suggesting that RON is implicated in endometriosis pathogenesis.^29^, ^30^ Recent studies have shown that RON activation can promote the migration and invasion of epithelial cancer cells in vitro.^31^, ^32^, ^33^ Endometriosis is a benign disease, but it has malignant biological behaviour, including proliferation, migration and invasion.^34^ Nevertheless, whether RON contributes to the development and progression of endometriosis by promoting EMT merits investigation.

We hypothesized that RON is involved in endometriosis pathogenesis by promoting the EMT of endometrial cells. First, we measured RON expression in endometriotic lesions from ovarian endometrioma. Second, we induced RON expression in endometrial cells in vitro. Third, we detected the effect of BMS 777607 (RON inhibitor) on endometriosis development in mice. In so doing, we explored the role of RON in endometriosis.

## 2 MATERIALS AND METHODS

### 2.1 Ethics statement

The scheme of this study was approved by the Human Ethics Committee of the Women's Hospital, School of Medicine, Zhejiang University in Zhejiang, China (approval number: 20160115). All patients gave written informed consent for their participation.

Animal experiments were carried out according to programmes approved by the ethics committee of Zhejiang University (NO. ZJU20190127).

### 2.2 Collection of clinical samples

Twenty‐eight patients with an ovarian endometrioma (seven women provided their ectopic endometria, twelve women provided their eutopic endometria and nine women provided both of their ectopic and eutopic endometria) and thirteen women without endometriosis who provided control endometria were recruited from Women's Hospital (Zhejiang University, School of Medicine). Patients with endometriosis had mainly a late‐stage (III and IV) ovarian endometrioma. All recruited women had regular menstrual cycles and none had not received any hormonal treatments for ≥3 months at the time of surgery. The endometrial samples used in this study were in the proliferative phase of the menstrual cycle, which was confirmed by the last menstrual period and endometrial histopathology.

### 2.3 Immunohistochemical (IHC) staining

Endometrial tissues were preserved in 4% formaldehyde and embedded in paraffin. Then they were cut into sections (thickness = 3 μm) using a microtome. After dewaxing, sections were incubated with antibodies to measure expression of RON and EMT markers in endometrial tissues: anti‐RON (1:600; ab52927; Abcam), anti‐Par3 (1:500; ab64840; Abcam), anti‐N‐cadherin (1:200; ab18203; Abcam), anti‐E‐cadherin (1:3000; 3195S; Cell Signaling Technology) and anti‐Occludin (1:500; ab216327; Abcam) to detect RON levels and EMT markers expression in endometrial tissues. The results of IHC staining were examined by two independent observers unaware of the sample's background. IHC scores were utilized based on the previously studies,^35^, ^36^ 0 represented no staining; one represented staining of 1%‐25%; two represented staining of 25%‐50%; three represented staining of 50%‐100%. Also, staining intensity was graded: zero represented no staining, one denoted ‘weak’ staining, two reflected ‘moderate’ staining, and three represented ‘high’ staining. The sum of the percentage score and the intensity score was represented as the immunostaining score. Finally, expression of these proteins was defined ‘low’ (score of 0‐5) or ‘high’ (score of 5‐6).

### 2.4 Culture of human endometrial cells and intervention

Isolation of primary human endometrial epithelial cells was undertaken as described in our previous paper.^37^ Then, cells were cultured in Dulbecco's Modified Eagle's medium: Nutrient Mixture F‐12 (DMEM/F 12; Keyi Biotechnology) containing penicillin (50 U/mL), streptomycin (50 μg/mL) and foetal bovine serum (FBS; 12% (v/v)), all of which were from Sigma‐Aldrich. The primary human endometrial epithelial cells we used were isolated from the control endometria in the proliferative phase and named ‘control endometrial epithelial cells’ (CEECs).

Cells were serum‐starved (0.5% FBS) for 24 hours before addition of BMS 777607 (Selleck Chemicals) or recombinant human MSP (R & D Systems).

### 2.5 Migration assays and invasion assays

We employed 24‐well Transwell™ plates (pore size, 8 μm; diameter, 6.5 mm) to carry out assays on migration and invasion assays. Human endometrial epithelial cells pre‐treated with MSP (200 ng/mL) or BMS 777607 (1 μmol/L) for 24 hours were resuspended in 200 μL of culture medium without serum, and added to the upper chambers (5 × 10^4^). Then 500 μL of culture medium containing 12% FBS was added to the bottom well and incubated at 37°C. After 24 hours of incubation, the cells were fixed and stained with crystal violet. Then a cotton swab was used to remove the cells that had not migrated into the lower chamber. Five randomly selected fields of each sample were counted under a light microscope, and each experiment was undertaken in triplicate. The invasion assay was carried out in a similar fashion except that the upper side of the filter was covered with Matrigel™ (#356234; BD Biosciences) before cells were seeded on the upper chamber.

### 2.6 Real‐time quantitative polymerase chain reaction (RT‐qPCR)

Total RNA was extracted using TRIzol™ Reagent (TaKaRa Biotechnology) and PrimeScript™ Reverse Transcription Reagent kit (TaKaRa Biotechnology) according to the manufacturer's protocols. RT‐qPCR was carried out using the SYBR Premix Ex Taq kit (TaKaRa Biotechnology). The primers used for amplification were synthesized from Generay Biotechnology and the sequences are listed: 5′‐ACTATGCCCACCGACCCT‐3′ and 5′‐GGTCGGTATGGATGGCGA‐3′ for N‐cadherin, 5′‐ATTTTTCCCTCGACACCCGAT‐3′ and 5′‐TCCCAGGCGTAGACCAAGA‐3′ for E‐cadherin, 5′‐GCGATGGTCATGCAGTCAG‐3′ and 5′‐CAGGTGGCAGGTCATTTTCTT‐3′ for ZEB2, 5′‐GTCAAGGCTGAGAACGGGAA‐3′ and 5′‐AAATGAGCCCCAGCCTTCTC‐3′ for GAPDH. Independent experiments were repeated thrice. Relative quantification of gene expression was achieved using the comparative 2^−ΔΔCT^ method.

### 2.7 Western blotting

RIPA buffer (Beyotime) was used to lyse cells on ice. The total concentrations of protein were determined by a BCA Protein Assay kit (#23227; Thermo). Equal amounts of protein were separated by sodium dodecyl sulphate‐polyacrylamide gel electrophoresis and then transferred to the polyvinylidene difluoride (PVDF) membranes (IPVH00010, Merck Millipore). After blockaded with 5% bovine serum albumin, PVDF membranes were incubated with anti‐E‐cadherin antibody (1:1000; 3195S; Cell Signaling Technology), anti‐N‐cadherin antibody (1:1000; ab18203; Abcam), anti‐ZEB2 antibody (1:1000; sc‐271984; Santacruze), anti‐protein kinase B (Akt) antibody (1:2000; 2920S; Cell Signaling Technology), anti‐P‐Akt antibody (1:1000; 4060S; Cell Signaling Technology), anti‐Par‐3 antibody (1:1000; ab64840; Abcam), anti‐P44/42 mitogen‐activated protein kinase (MAPK) antibody (1:1000; 4695S; Cell Signaling Technology), anti‐P‐P44/42 MAPK antibody (1:1000; 4376S; Cell Signaling Technology), anti‐RON β antibody (1:500; sc‐25781; Santa Cruz Technology), anti‐P‐RON antibody (1:1000; AF1947; R & D Systems) and anti‐glyceraldehyde 3‐phosphate dehydrogenase (GAPDH) antibody (1:1000; A95370123; Multisciences Biotech) overnight at 4°C. After incubated with horseradish peroxidase‐conjugated secondary antibody against rabbit immunoglobulin (Ig)G (1:10 000, ab97051, Abcam) or against mouse IgG (1:10 000, ab97023, Abcam), the PVDF membranes were detected by an electrochemiluminescence detection kit (Biological Industries) according to the manufacturer specifications. Independent experiments were repeated three thrice and protein expression was quantified based on band volume using Image J software (National Institutes of Health) and normalized with respect to GAPDH expression.

### 2.8 Transfection of small interfering (si) RNA

Three siRNAs targeted to the RON (siRON) gene were purchased from GenePharma. CEECs were seeded onto 12‐well plates and transfected with siRON (10 pmol/L) or negative control using LipofectamineTM RNAiMAX (Invitrogen) according to the manufacturer's protocols. Cells were used for migration assays and invasion assays after 48 hours of transfection, and cells transfected for 96 hours were used for Western blotting.

### 2.9 Animals and treatment

Eighteen female BALB/c mice, 6**‐**8 weeks, were used in this research. The model of endometriosis was induced as described by Somigliana and colleagues.^38^ All mice underwent ovariectomy and were injected with estradiol benzoate (500 ng/mouse/5 d, s.c.) 1 week before endometriosis induction. Six mice were selected randomly as donors; their uteri horns were harvested and placed into sterile physiologic (0.9%) saline. Then their uteri horns were split longitudinally and minced into small fragments (<1 mm^3^) with a pair of scissors. Endometrial‐tissue fragments obtained from one mouse were suspended in 1.2 mL of sterile 0.9% saline and injected (i.p.) into two recipient mice.

One day after endometriosis induction, mice were divided randomly into three groups of four. Then mice were treated with vehicle control (dimethyl sulphoxide:polyethylene glycol 400:water = 3:70:27), BMS 777607 (15 mg/kg) or BMS 777607 (30 mg/kg) by intraperitoneally injection once daily. After 21 days of treatment, the mice were sacrificed, and endometric lesions were removed carefully. The number of endometriotic lesions was counted, the weight of endometriotic lesions was measured, and the volume of a single endometriotic lesion was calculated using the formula (volume = 0.5 × length × width^2^). Then the endometrial tissues were preserved in 4% formaldehyde and embedded in paraffin, and cut into 3 μm‐thick sections using a microtome. Next, the sections were examined by IHC staining. The IHC procedures were similar to those described above, except for the antibodies: anti‐N‐cadherin (1:800; ab18203; Abcam), anti‐E‐cadherin (1:200; 3195S; Cell Signaling Technology) and anti‐RON β (1:250; sc‐25781; Santacruze).

### 2.10 Statistical analyses

Statistical analyses were carried out using IBM SPSS 17.0 (IBM). The Student's *t* test was used to determine the differences between two measurements. One‐way ANOVA analysis or Kruskal's test was employed to evaluate the multiple comparisons. Fisher's exact test was used to analyse the categorical variables. *P* < .05 was considered significant.

## 3 RESULTS

### 3.1 RON was overexpressed in ovarian endometriotic lesions

Previously, we revealed that RON was overexpressed in secretory ovarian endometriotic lesions.^30^ In this study, we investigated the RON expression in control endometria (n = 13), eutopic endometria (n = 21) and ovarian endometriotic lesions (n = 16), and all of them were proliferative endometria.

**FIGURE 1:**
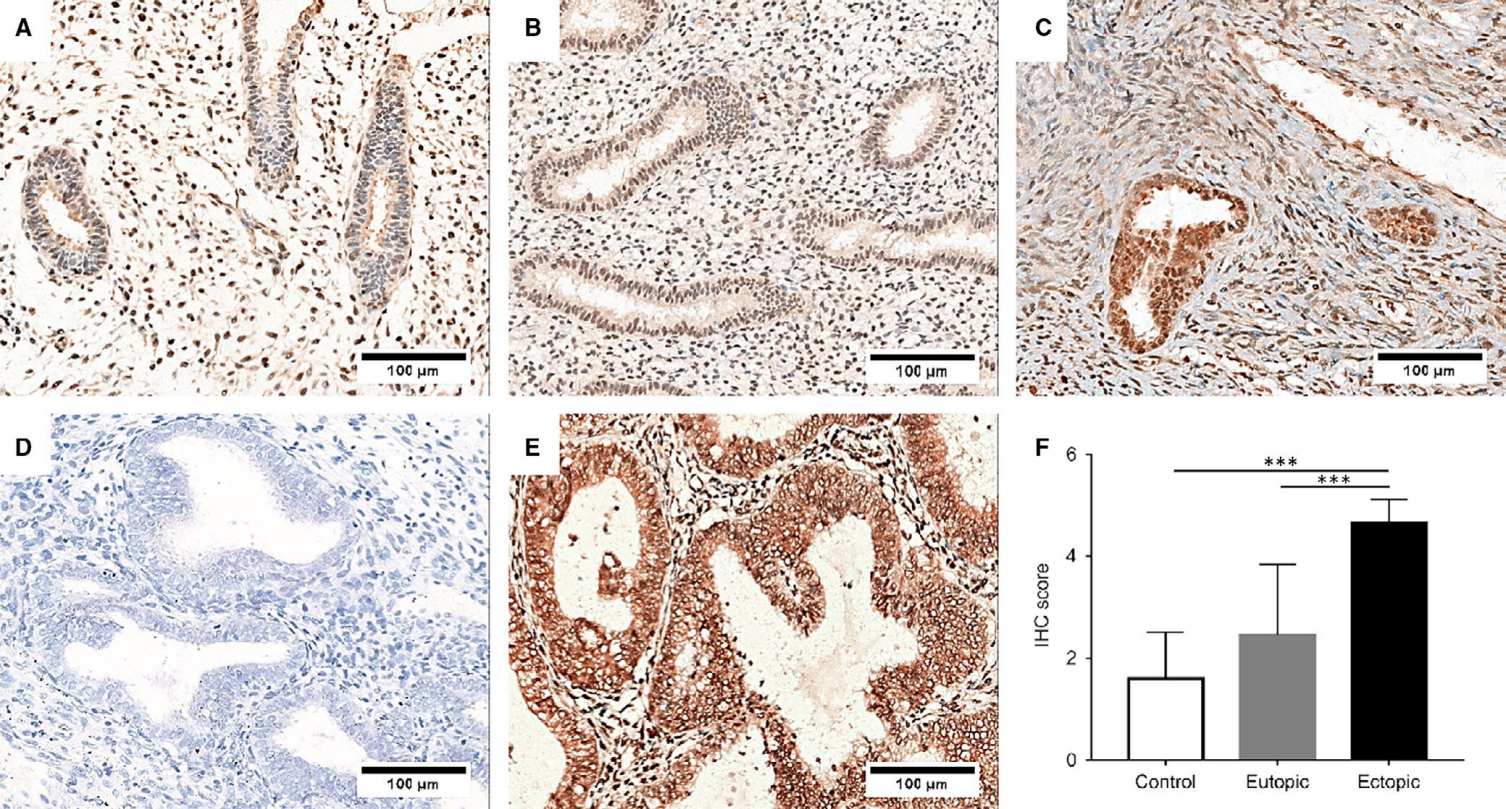
RON was overexpressed in ovarian endometriotic lesions. Immunohistochemical (IHC) staining was performed to detect the expression of RON in control endometria (A), eutopic endometrial tissues (B) and ovarian endometriotic lesions (C), D, negative control without primary antibody, E, endometrial carcinoma was used as a positive control for RON, the scale bars represent 100 µm. F, the immunostaining score of RON in three different endometrial tissues. The data were represented as mean ± SD, ***P < .001. N = 13 for control group, N = 21 for eutopic group and N = 16 for ectopic group

Immunohistochemical staining showed that the RON expression in ovarian endometriotic lesions was higher than that in control and eutopic endometrial tissues in the proliferative phase (Figure 1). The score of RON expression in ovarian endometriotic lesions (4.66 ± 0.45) was significantly higher than that in control and eutopic endometrial tissues. These results illustrated that RON was overexpressed in ovarian endometriotic lesions and that it may be involved in the development of ovarian endometrioma.

### 3.2 EMT occurs in endometrial epithelial cells of ovarian endometriotic lesions

**FIGURE 2:**
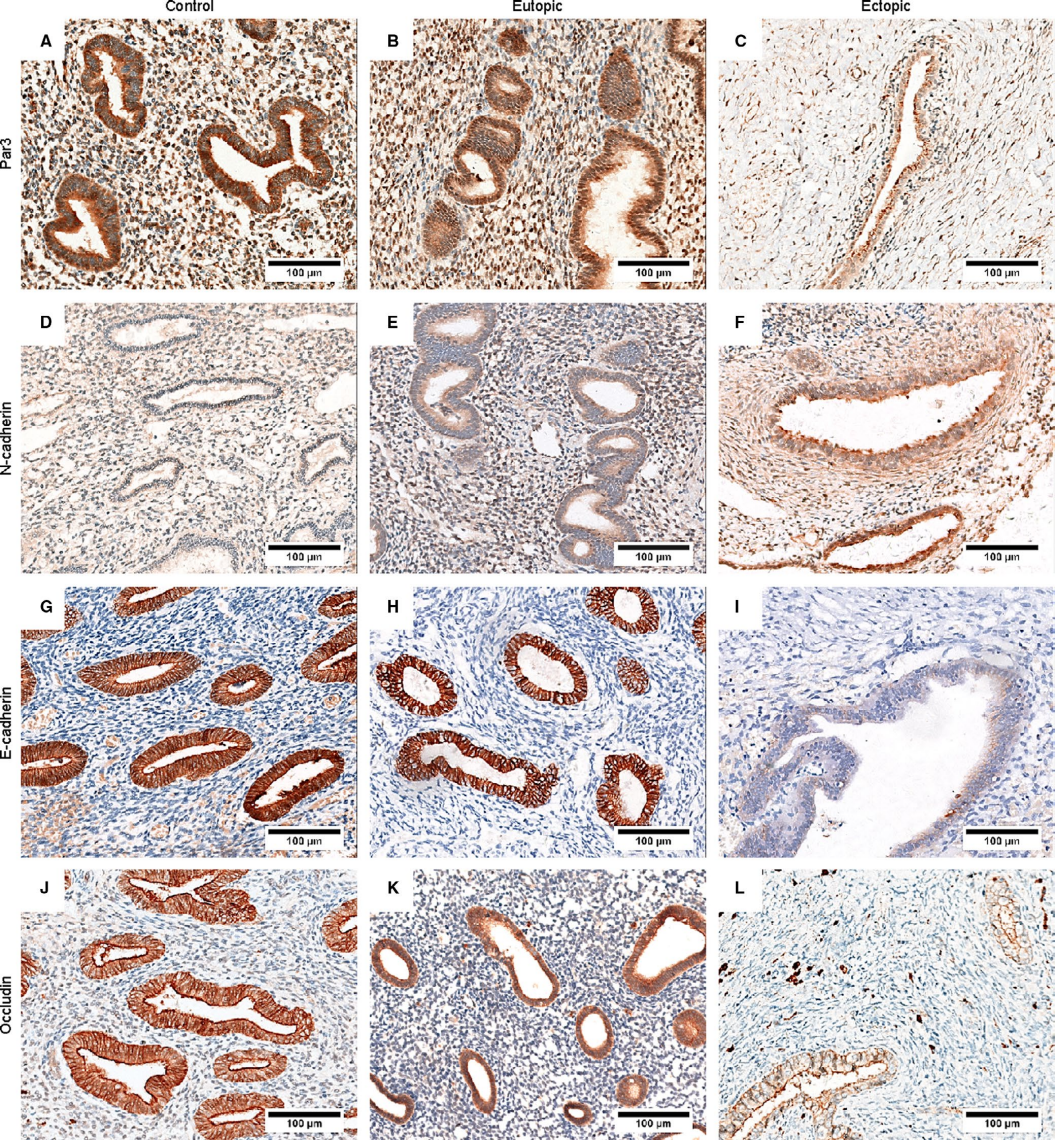
EMT occurs in endometrial epithelial cells of ovarian endometriotic lesions. The representative photomicrographs of Par3 (A‐C), N‐cadherin (D‐F), E‐cadherin (G‐I) and Occludin (J‐L). The scale bars represent 100 µm

Several scholars have been proposed that EMT participates in endometriosis.^9^ To investigate whether EMT occurs in ovarian endometrioma, the expression of EMT markers was assessed by IHC staining in control endometria (n = 13), eutopic endometria (n = 21) and ovarian endometriotic lesions (n = 16). The IHC results are illustrated in Figure 2, and expression profile of the EMT markers in endometrial tissues and the IHC score is displayed in Table 1.

**TABLE 1:** Expression profile and IHC score of the EMT markers in endometrial tissues

	Control group 1	Eutopic group 2	Ectopic group 3	*P*‐value 1 vs 2	*P*‐value 2 vs 3	*P*‐value 1 vs 3
Par3						
Low	10 (76.9%)	21 (100%)	16 (100%)	.048*	—	.078
High	3 (23.1%)	0	0			
N‐cad						
Low	13 (100%)	21 (100%)	6 (37.5%)	—	<.001***	<.001***
High	0	0	10 (62.5%)			
E‐cad						
Low	2 (15.4%)	1 (4.8%)	13 (81.2%)	.544	<.001***	<.001***
High	11 (84.6%)	20 (95.2%)	3 (18.8%)			
OCLN						
Low	1 (7.7%)	7 (33.3%)	14 (87.5%)	.116	.002**	<.001***
High	12 (92.3%)	14 (66.7%)	2 (12.5%)			
IHC score (Mean ± SD)						
Par3	5.00 ± 0.36	4.45 ± 0.76	3.96 ± 0.59	.007**	.041*	<.001***
N‐cad	3.41 ± 0.73	3.23 ± 0.79	5.29 ± 0.26	.496	<.001***	<.001***
E‐cad	5.51 ± 0.58	5.64 ± 0.31	4.64 ± 0.39	.421	<.001***	<.001***
OCLN	5.68 ± 0.32	5.19 ± 0.39	4.89 ± 0.41	.001**	.03*	<.001***

Abbreviations: E‐cad, E‐cadherin; N‐cad, N‐cadherin; OCLN, Occludin.

* *P* < .05.

** *P* < .01.

*** *P* < .001.

Par3 is an important protein for maintaining apical‐basal polarity, which is first lost during EMT.^39^ Epithelial cells of control endometria presented the highest staining of Par3 compared with that of eutopic and ectopic endometria (Figure 2, Table 1). The IHC score of ectopic endometria was lower than that of eutopic endometria (Table 1).

Strong staining of N‐cadherin was found in 62.5% of endometriotic lesions. Only weak staining appeared in control and eutopic endometrial tissues, and the IHC score of endometriotic lesions was also the highest among them (Figure 2, Table 1). In contrast, strong staining of E‐cadherin was detected in most of control and eutopic endometrial tissues (84.6% and 95.2%). Only 18.8% of ectopic endometria showed strong staining (Figure 2, Table 1) and their IHC score was lower than that of control and eutopic endometrial tissues.

Serving as a tight junction protein for epithelial cells, Occludin exhibited similar staining to that observed for E‐cadherin. Only 12.5% of endometriotic lesions had strong staining, whereas the percentage of strong staining in control and eutopic endometria were 92.3% and 66.7% respectively (Figure 2, Table 1). Taken together, these data explained that the epitheliums of ovarian endometriotic lesions had gone through the EMT.

### 3.3 RON induced EMT in endometrial epithelial cells

**FIGURE 3:**
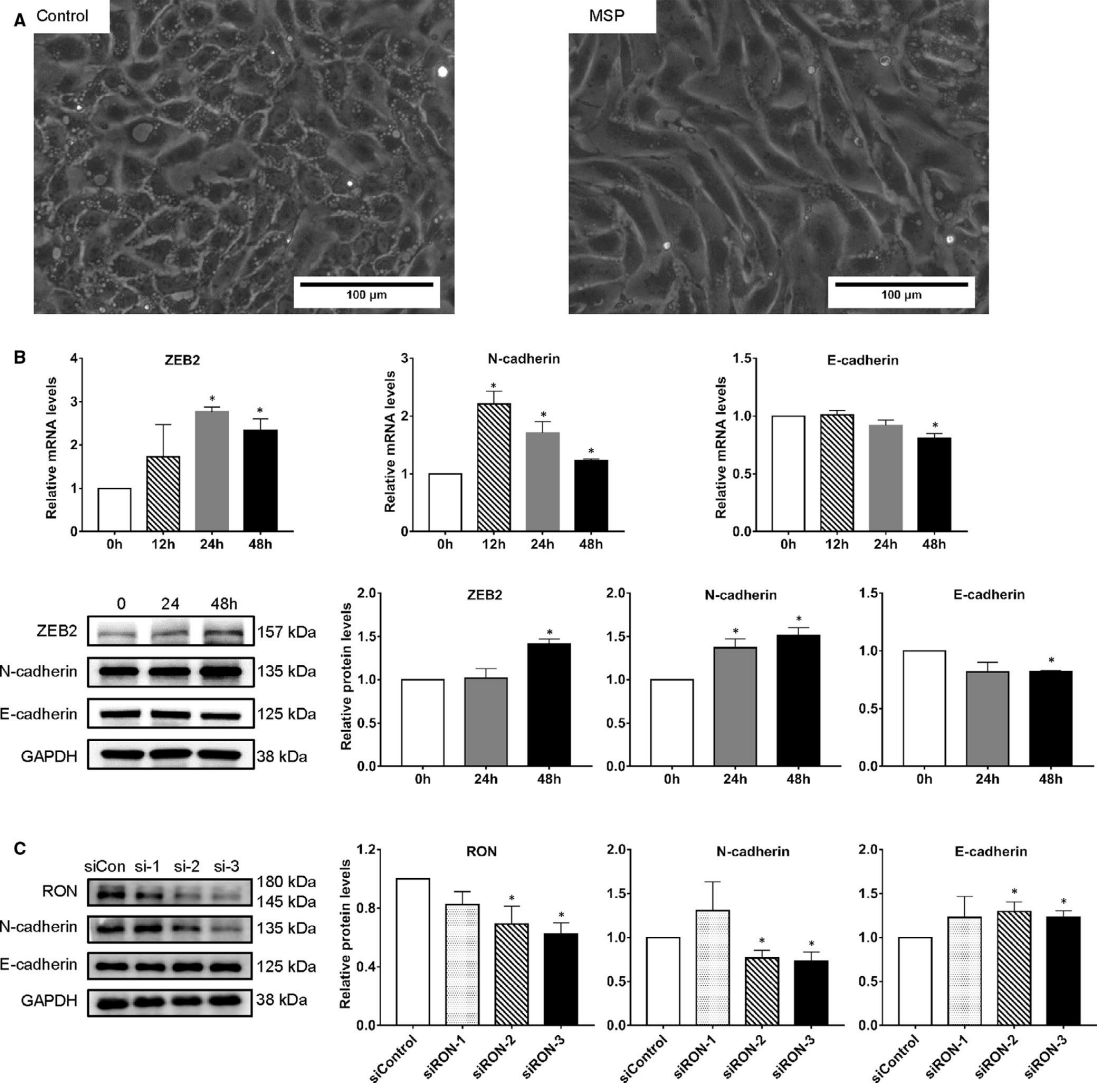
RON induces EMT in endometrial epithelial cells. A, morphology of primary endometrial epithelial cells changed after stimulated with MSP for 48 h. The scale bars represent 100 µm. B, expression of EMT markers in primary endometrial epithelial cells after MSP stimulation. C, expression of EMT markers in primary endometrial epithelial cells transfected with siRON for 96 h. Values represent the mean ± SD, *P < .05. All of the assays were repeated three times

Based on the data mentioned above, we realized that RON expression increased and EMT occurred in epithelial cells of ovarian endometrioma samples. RON has been reported to promote EMT in tumour metastasis,^31^ so we speculated that RON could induced EMT in endometrial epithelial cells. Spindle‑like changes in cell morphology are an important feature of EMT, and we observed the changes in morphology of primary CEECs exposed to MSP (200 ng/mL) for 48 hours. The cells changed from cubes to spindles, the contacts between cells tend to be lost and cell clusters were scattered (Figure 3A), which illustrated that the cells had undergone the morphological changes seen typically in EMT. Next, we measured expression of EMT markers in CEECs. Expressions of N‐cadherin and ZEB2 were increased, whereas expression of E‐cadherin was reduced, in MSP treated cells (Figure 3B).

To further evaluate the effect of RON on endometrial epithelial cells, we used siRON to transfected CEECs. Expression of N‐cadherin protein was down‐regulated by siRON and that of E‐cadherin protein level was up‐regulated (Figure 3C).

### 3.4 RON promotes the migration and invasion of endometrial epithelial cells

Enhanced migration and invasion capabilities are a major feature of EMT and RON has been demonstrated to play an important part in the migration and invasion of malignant tumour cells, so we speculated that RON could enhance the migratory and invasive capabilities of endometrial epithelial cells by promoting EMT. We evaluated the effects of MSP (agonist of RON) and BMS 777607 (inhibitor of RON) on the migration and invasion of CEECs.

**FIGURE 4:**
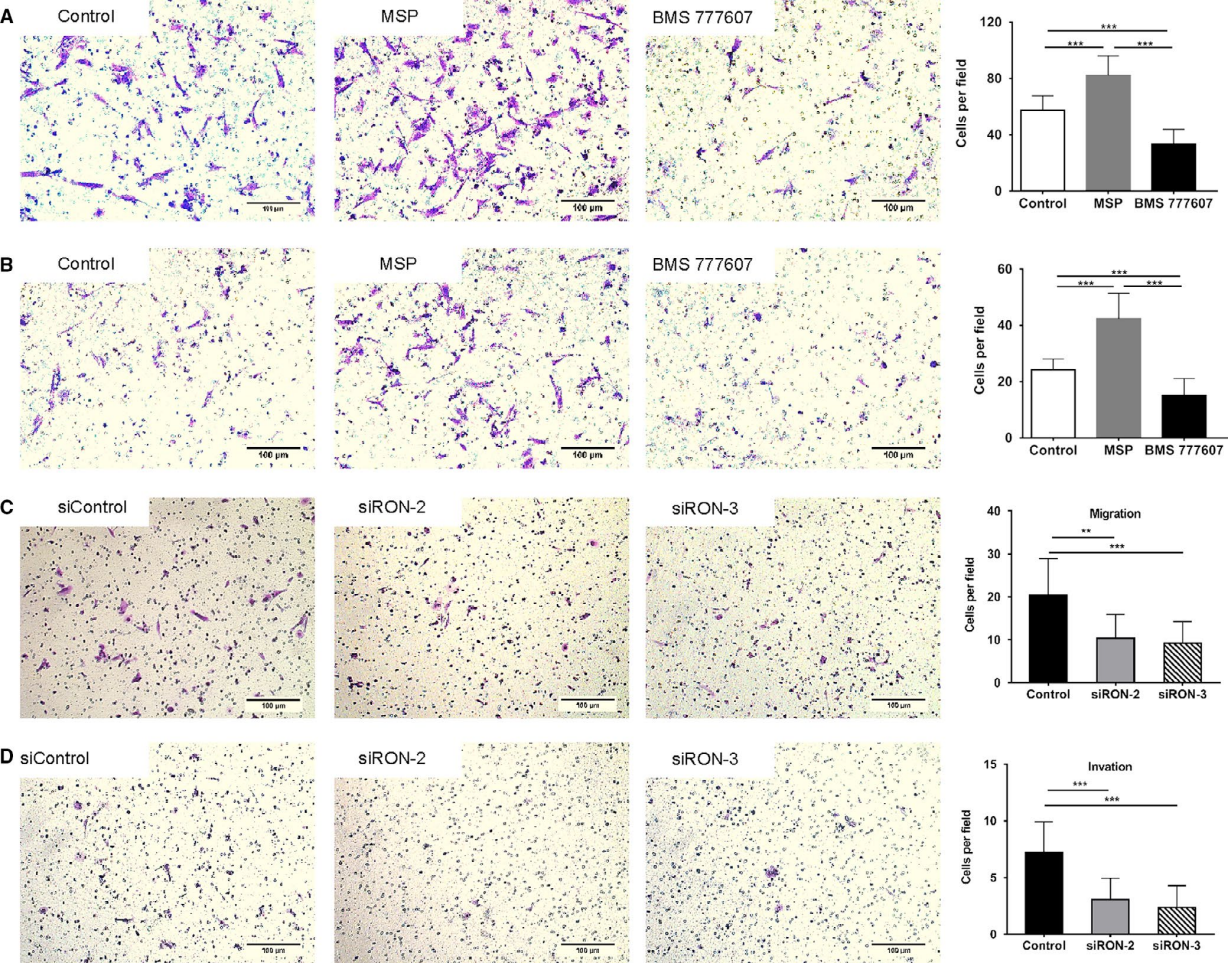
RON promotes the migration and invasion of endometrial epithelial cells. A, representative photomicrographs of primary control endometrial epithelial cells migration after treated with MSP or BMS 777607 for 24 h. B, representative photomicrographs of primary control endometrial epithelial cells invasion treated with MSP or BMS 777607 for 24 h. C, representative photomicrographs of primary endometrial epithelial cells migration transfected with siRON for 48 h. D, representative photomicrographs of primary endometrial epithelial cells invasion transfected with siRON for 48 h. The scale bars represent 100 µm. Values represent the mean ± SD, *P < .05, ***P < .001. All of the assays were repeated thrice

Upon stimulation with MSP, the number of CEECs that migrated was 82 ± 14, which was increased significantly compared with control group (57 ± 10), whereas the number of cells was decreased significantly after treated with BMS 777607 (33 ± 10) (Figure 4A). Similar results were seen in the invasion assay: MSP could enhance the invasion of CEECs remarkably, and BMS 777607 inhibited this process (Figure 4B). Transfection with RON siRNA could also reduce the migration and invasion ability of CEECs (Figure 4C,D). These data shown above suggested that RON increased the migration and invasion of endometrial epithelial cells through the promotion of EMT in endometriosis.

### 3.5 RON promoted EMT through Akt and MAPK signalling pathways in human endometrial epithelial cells

**FIGURE 5:**
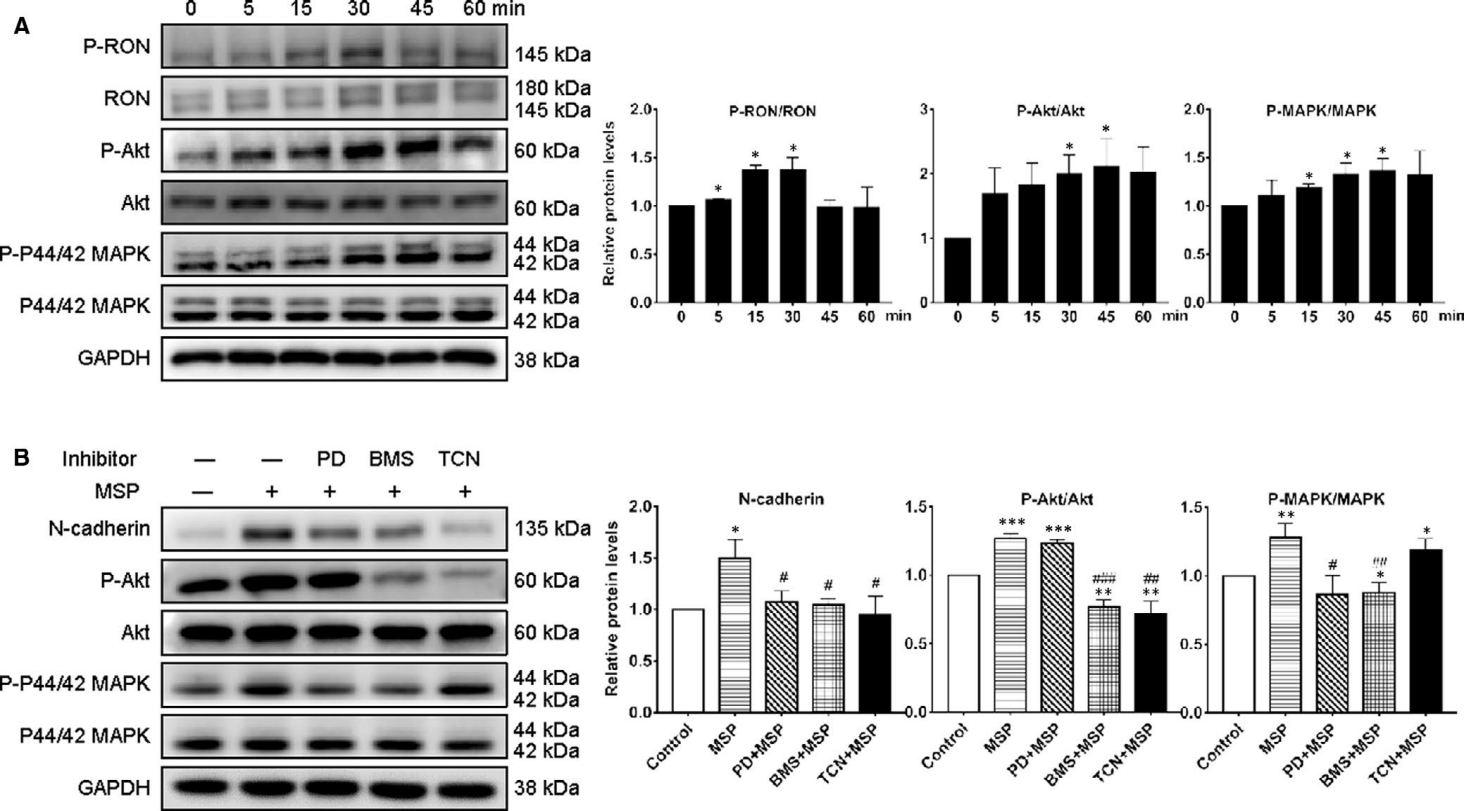
RON promoted EMT through Akt and MAPK signalling pathways in human endometrial epithelial cells and inflammatory cytokines participate in this process. A, MSP treatment increased the phosphorylation of RON, P44/42 MAPK and Akt in control endometrial epithelial cells. Three times experiments were repeated and the values were expressed as mean ± SD (*P < .05, **P < .01, compared with control group). B, the effects of inhibitors on MSP‐induced EMT (BMS, BMS777607; PD, PD98059; TCN, triciribine), three times experiments were repeated and the values were expressed as mean ± SD (*P < .05, **P < .01, ***P < .001 compared with control group, ^#^ *P* < .05, ^##^ *P* < .01, ^###^ *P* < .001 compared with MSP group). Three times experiments were repeated and the values were expressed as mean ± SD (*P < .05, **P < .01, ***P < .001 compared with control group)

Macrophage‐stimulating protein could induce EMT in human endometrial epithelial cells and enhance the migration and invasiveness of CEECs, but the signalling involved in these changes is not known. Recent cancer research has suggested that RON participates in the biological processes of tumours mainly through RAS‐MAPK and phosphoinositide 3‐kinase (PI3K)/AKT pathways.^33^ Then Western blotting was performed on CEECs stimulated with MSP to evaluate the downstream signalling activated by RON. MSP could phosphorylate RON, Akt and P44/42 MAPK (Erk) significantly, with peak activation at 15‐30 minutes for RON and 30‐45 minutes for Akt and P44/42 MAPK (Figure 5A), indicating that RON could act on endometrial cells through these two pathways.

To further determine whether these pathways were associated with EMT, we investigated the effects of PD98059 (Erk inhibitor, APExBIO), triciribine (Akt inhibitor, MedChemExpress) and BMS 777607 on MSP‐induced EMT, respectively. After CEECs had been treated with PD98059 (10 μmol/L), triciribine (10 μmol/L) or BMS 777607 (10 μmol/L) for 10 minutes and then stimulated with MSP (200 ng/mL) for 30 minutes, CEECs were collected and analysed by Western blotting. PD98059, BMS 777607 and triciribine had a significant inhibitory effect on the increase in N‐cadherin expression induced by MSP (Figure 5B). Taken together, these data suggested that RON may promote EMT in endometriosis through Akt and MAPK pathways.

### 3.6 RON inhibitor suppressed the development of endometriosis via the restriction of EMT in vivo

**FIGURE 6:**
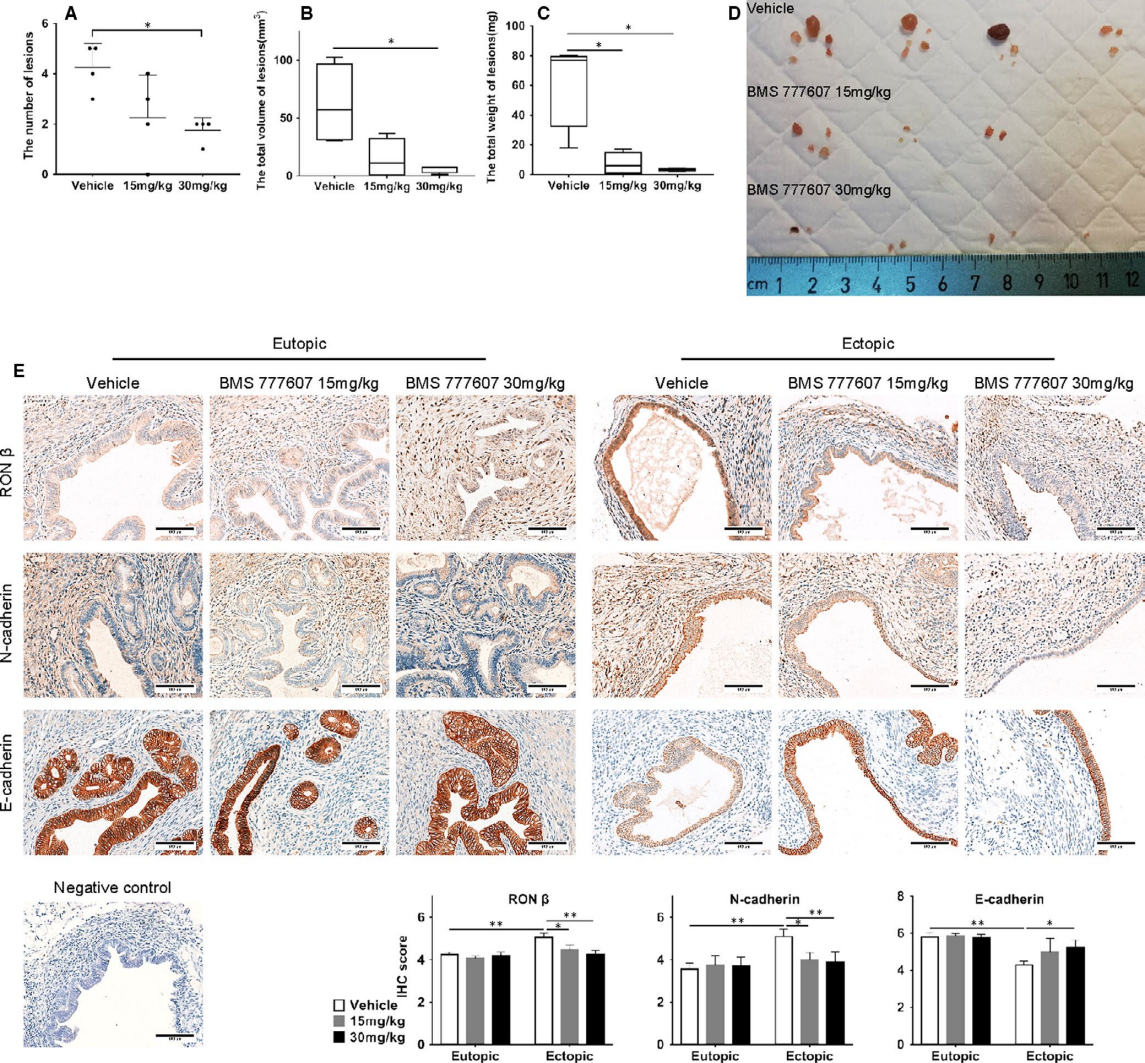
Effect of BMS 777607 (RON inhibitor) on endometriosis development in vivo. A, number of endometriotic lesions from different groups of mice. B, total volume of endometriotic lesions from each mice in different groups. C, total weight of endometriotic lesions from each mice in different groups. D, general appearance of endometriotic lesions from mice of different groups. E, immunostaining results of RON, N‐cadherin and E‐cadherin in eutopic endometrial tissues and endometriotic lesions from different groups of mice. The scale bars represent 100 µm. N = 4 for each group. *P < .05, **P < .01

To further study the role of RON in endometriosis, we constructed a mouse model of endometriosis and undertook treatment with the RON inhibitor BMS 777607. Compared with the control group, treatment with BMS 777607 could reduce the number of ectopic lesions in endometriosis‐model mice, and the volume and weight of ectopic lesions were also reduced (Figure 6A‐D). IHC staining showed that, in the control group, the expression of RON and N‐cadherin in endometriotic lesions increased and that of E‐cadherin decreased, compared with that in eutopic endometria. These findings further demonstrated that EMT occurred in endometriotic lesions. BMS777607 treatment could reverse EMT process in the ectopic tissue (Figure 6E). These results further suggested that RON participates in endometriosis development by inducing EMT in endometrial cells and enhancing their migration and invasion abilities, which can be inhibited by BMS 777607.

## 4 DISCUSSION

We discovered that both RON and EMT are associated with endometriosis development, and that RON can promote migratory and invasive abilities and induce EMT in human endometrial epithelial cells. The role of RON in endometriosis development was confirmed further by studying its agonist and inhibitor.

Previously, we showed RON expression in ovarian endometriotic lesions to be significantly higher than that in peritoneal and deeply infiltrating endometriotic lesions.^30^ We speculated that the role of RON played in ovarian endometrioma may be greater, so we focused mainly on ovarian endometrioma in the present study. Importantly, due to the use of different antibodies purchased from different companies, the IHC score of RON in the present study was greater than that in our previous study, but the trend of RON expression in different tissues was consistent with that in our previous study.

Epithelial**‐**mesenchymal transition includes a loss of polarity, damage to cell adhesion and the acquisition of migratory ability.^13^ We used N‐cadherin, E‐cadherin, Occludin and Par3 as markers to study EMT of ovarian endometrioma. N‐cadherin expression was elevated in ovarian ectopic lesions compared with that in control and eutopic tissues, whereas expression of E‐cadherin, Par3 and Occludin expression was reduced, indicating that EMT was involved in the development of ovarian endometrioma. In vitro, we found that MSP stimulation resulted in changes in EMT‐like morphology and expression of EMT markers expression in CEECs. siRON could reduce expression of mesenchymal markers in endometrial epithelial cells. We showed, in a preliminarily manner, that RON could induce EMT in endometrial cells. It is worth noting that compared with N‐cadherin, the change of E‐cadherin expression due to RON is less. The role of E‐cadherin in endometriosis is controversial: some studies have displayed its expression to be decreased in endometriosis,^40^, ^41^ but others have denied such a decrease or even shown an increase.^19^, ^42^, ^43^, ^44^ Repression of E‐cadherin transcription has been considered as a key step of EMT for many years, but an increasing number of studies has shown that the role of E‐cadherin in EMT is complicated. Recently, the concept of an ‘EMT spectrum’ has been proposed,^45^, ^46^, ^47^ whereby EMT is a series of graded processes with intermediate EMT. Intermediate EMT, also known as ‘partial EMT’, is the intermediate stage of EMT, and the characteristics of the epithelium and mesenchyme coexist.^48^ In addition, some studies have pointed out that high expression of E‐cadherin in endometriotic glands may be due to endometriosis being a benign disease^21^ and the polymorphism of the E‐cadherin gene.^49^ Additional studies are needed to do to reveal the role of E‐cadherin in endometriosis.

Epithelial**‐**mesenchymal transition can promote invasion and metastasis, and we detected the effect of RON on the migratory and invasive abilities of endometrial epithelial cells. RON activation could promote migration and invasion of endometrial epithelial cells, whereas RON inhibition had the opposite effect, these results confirmed the role of RON in endometriosis.

RAS‐MAPK and PI3K/AKT are the main signalling pathways in the action of RON.^33^ Camp et al^50^ discovered that MSP can increase phosphorylation of Akt and Erk in L3.6pl cells that result is in accordance with our results in endometrial epithelial cells. Different from our results, two studies demonstrated that an inhibitor of Erk (PD98059) could prevent the EMT induced by MSP in MDCK cells while an inhibitor of PI3K (wortmannin) could not.^51^, ^52^ This difference may have been caused by use of different cells or inhibitor in the two studies.

We further explored the role of RON in endometriosis using a mouse model of endometriosis. Dai and colleagues reported that treatment with BMS‐777607 could reduce the number of KHT lung tumour nodules.^53^ Similar results were obtained in our study: BMS 777607 had an inhibitory effect on the development of ectopic lesions and reversed the EMT phenomenon of ectopic lesions in vivo.

In conclusion, we demonstrated that RON contributes to the development and progression of endometriosis. In vitro and in vivo studies proved that RON could promote the migration, invasion and induce EMT of endometrial epithelial cells. We showed that RON may mediate EMT through RAS‐MAPK and PI3K/AKT pathways in endometrial epithelial cells. More research is needed to discover the key molecules that RON regulates during EMT in endometriosis, and to find better treatments for endometriosis.

## CONFLICT OF INTEREST

The authors have declared that no competing interest exists.

## AUTHOR CONTRIBUTION

**Qin Yu:** Data curation (lead); Formal analysis (lead); Investigation (lead); Methodology (lead); Writing‐original draft (lead); Writing‐review & editing (equal). **Jianzhang Wang:** Data curation (equal); Formal analysis (equal); Writing‐original draft (supporting); Writing‐review & editing (equal). **Tiantian Li:** Data curation (equal); Formal analysis (equal); Investigation (supporting). **Xinyue Guo:** Data curation (equal); Formal analysis (equal). **Shaojie Ding:** Data curation (equal); Formal analysis (supporting); Methodology (equal). **Xuan Che:** Data curation (supporting); Formal analysis (equal). **Libo Zhu:** Data curation (supporting); Formal analysis (equal); Investigation (supporting). **Yangying Peng:** Data curation (supporting); Formal analysis (supporting); Investigation (supporting). **Xinxin Xu:** Data curation (supporting); Formal analysis (supporting); Investigation (supporting). **Gen Zou:** Data curation (supporting); Formal analysis (supporting). **Xinmei Zhang:** Project administration (lead); Writing‐original draft (supporting); Writing‐review & editing (lead).

## Data Availability

The data that support the findings of this study are available from the corresponding author upon reasonable request.
